# Therapeutic Effects of Butyrate on Pediatric Obesity: A Randomized Clinical Trial

**DOI:** 10.1001/jamanetworkopen.2022.44912

**Published:** 2022-12-05

**Authors:** Serena Coppola, Rita Nocerino, Lorella Paparo, Giorgio Bedogni, Antonio Calignano, Carmen Di Scala, Anna Fiorenza de Giovanni di Santa Severina, Francesca De Filippis, Danilo Ercolini, Roberto Berni Canani

**Affiliations:** 1 Department of Translational Medical Science, University of Naples “Federico II,” Naples, Italy; 2 ImmunoNutritionLab, CEINGE-Advanced Biotechnologies, University of Naples “Federico II,” Naples, Italy; 3 Department of Medical and Surgical Sciences, Alma Mater Studiorum University of Bologna, Bologna, Italy; 4 Department of Primary Health Care, Internal Medicine Unit Addressed to Frailty and Aging, S Maria delle Croci Hospital, AUSL Romagna, Ravenna, Italy; 5 Department of Pharmacy, University of Naples “Federico II,” Naples, Italy; 6 Task Force on Microbiome Studies, University of Naples “Federico II,” Naples, Italy; 7 Department of Agricultural Sciences, University of Naples “Federico II,” Naples, Italy; 8 European Laboratory for the Investigation of Food-Induced Diseases, University of Naples “Federico II,” Naples, Italy

## Abstract

**Importance:**

The pediatric obesity disease burden imposes the necessity of new effective strategies.

**Objective:**

To determine whether oral butyrate supplementation as an adjunct to standard care is effective in the treatment of pediatric obesity.

**Design, Setting, and Participants:**

A randomized, quadruple-blind, placebo-controlled trial was performed from November 1, 2020, to December 31, 2021, at the Tertiary Center for Pediatric Nutrition, Department of Translational Medical Science, University of Naples Federico II, Naples, Italy. Participants included children aged 5 to 17 years with body mass index (BMI) greater than the 95th percentile.

**Interventions:**

Standard care for pediatric obesity supplemented with oral sodium butyrate, 20 mg/kg body weight per day, or placebo for 6 months was administered.

**Main Outcomes and Measures:**

The main outcome was the decrease of at least 0.25 BMI SD scores at 6 months. The secondary outcomes were changes in waist circumference; fasting glucose, insulin, total cholesterol, low-density lipoprotein cholesterol, high-density lipoprotein cholesterol, triglyceride, ghrelin, microRNA-221, and interleukin-6 levels; homeostatic model assessment of insulin resistance (HOMA-IR); dietary and lifestyle habits; and gut microbiome structure. Intention-to-treat analysis was conducted.

**Results:**

Fifty-four children with obesity (31 girls [57%], mean [SD] age, 11 [2.91] years) were randomized into the butyrate and placebo groups; 4 were lost to follow-up after receiving the intervention in the butyrate group and 2 in the placebo group. At intention-to-treat analysis (n = 54), children treated with butyrate had a higher rate of BMI decrease greater than or equal to 0.25 SD scores at 6 months (96% vs 56%, absolute benefit increase, 40%; 95% CI, 21% to 61%; *P* < .01). At per-protocol analysis (n = 48), the butyrate group showed the following changes as compared with the placebo group: waist circumference, −5.07 cm (95% CI, −7.68 to −2.46 cm; *P* < .001); insulin level, −5.41 μU/mL (95% CI, −10.49 to −0.34 μU/mL; *P* = .03); HOMA-IR, −1.14 (95% CI, −2.13 to −0.15; *P* = .02); ghrelin level, −47.89 μg/mL (95% CI, −91.80 to −3.98 μg/mL; *P* < .001); microRNA221 relative expression, −2.17 (95% CI, −3.35 to −0.99; *P* < .001); and IL-6 level, −4.81 pg/mL (95% CI, −7.74 to −1.88 pg/mL; *P* < .001). Similar patterns of adherence to standard care were observed in the 2 groups. Baseline gut microbiome signatures predictable of the therapeutic response were identified. Adverse effects included transient mild nausea and headache reported by 2 patients during the first month of butyrate intervention.

**Conclusions and Relevance:**

Oral butyrate supplementation may be effective in the treatment of pediatric obesity.

**Trial Registration:**

ClinicalTrials.gov Identifier: NCT04620057

## Key Points

**Question:**

Can oral butyrate supplementation as adjunct to standard care be effective in the treatment of pediatric obesity?

**Findings:**

In this randomized clinical trial including 54 children with obesity, a 40% absolute increase in the rate of children experiencing a decrease of at least 0.25 SD scores of body mass index was observed at 6 months in those treated with butyrate compared with those receiving placebo in an intention-to-treat analysis. Adverse effects included transient mild nausea and headache reported by 2 patients during the first month of butyrate intervention.

**Meaning:**

The findings of this randomized clinical trial suggest that oral butyrate supplementation may be effective in the treatment of pediatric obesity.

## Teaser

This randomized clinical trial examines the use of butyrate supplementation in addition to standard care in children with obesity.

## Introduction

Pediatric obesity is growing at an alarming rate worldwide.^1,2^ Effective therapeutic strategies are needed to limit the disease burden.

Gut microbiome (GM) could play a role in obesity.^3,4,5^ A metabolically healthy GM is maintained by a diet rich in fiber.^6^ Plant foods are fermented by GM to produce the antiobesogenic short-chain fatty acid butyrate.^7,8^ Although butyrate dietary intake could be increased by the consumption of dairy products, its main source is derived by GM fermentation of nondigestible carbohydrates.^8^ A low intake of dietary substrates for butyrate production and a low number of butyrate-producing bacteria may contribute to obesity.^9^ This evidence suggests the potential of butyrate for treating obesity. The Butyrate Against Pediatric Obesity (BAPO) trial was designed to evaluate whether butyrate supplementation can be effective in pediatric obesity treatment.

## Methods

### Trial Design

**Figure 1.**
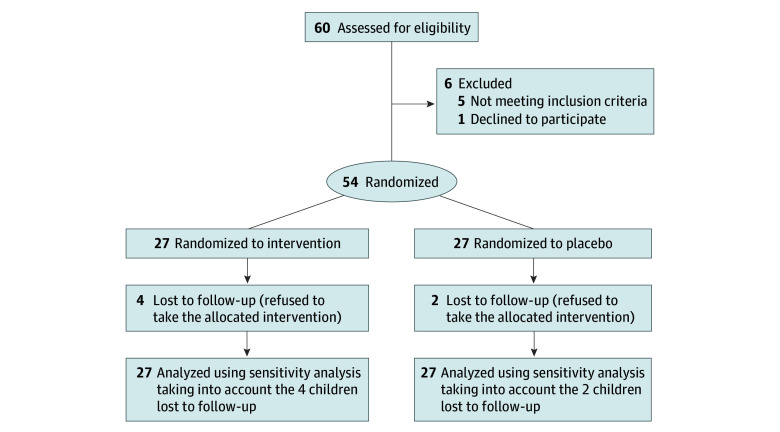
Participants Recruited and Randomized in the Butyrate Against Pediatric Obesity Trial

The BAPO trial was a randomized, quadruple-blind, parallel-group, placebo-controlled trial aimed at testing the therapeutic efficacy of butyrate supplementation in pediatric patients with obesity. The trial was approved by the ethics committee of the University of Naples “Federico II” (protocol available in Supplement 1) and was performed in accordance with the Helsinki Declaration^10^ and with the relevant European and Italian privacy regulations. Written informed consent to participate in the study was obtained from the patients’ parents or legal guardian. No financial compensation was provided. This study followed the Consolidated Standards of Reporting Trials (CONSORT) reporting guideline (Figure 1).

### Participants

Patients followed up at the Tertiary Center for Pediatric Nutrition of the Department of Translational Medical Science at the University of Naples “Federico II” were considered eligible for the study. Inclusion criteria were age 5 to 17 years and body mass index (BMI) greater than the 95th percentile for sex and age, using the World Health Organization growth charts.^11^ We excluded children with allergies, autoimmune diseases, bariatric surgery, cancer, celiac disease, chronic hematologic diseases, cystic fibrosis, gastrointestinal diseases, respiratory diseases, urinary tract diseases, genetic and metabolic diseases, immunodeficiencies, inflammatory bowel diseases, neurologic or neuropsychiatric disorders, participation in other trials, and treatment with metformin, vitamins, prebiotics, probiotics, or postbiotics because of their potential effect on GM structure and function in the past year.

### Intervention

The butyrate group received standard care for pediatric obesity plus sodium butyrate capsules, 20 mg/kg body weight per day, up to a maximum of 800 mg/d (Butyrose LSC microcaps) for 6 months (T6). The placebo group received standard care plus cornstarch capsules. In the instance that younger patients were unable to swallow capsules, children were invited to open the capsules and dissolve the content in liquid or solid foods. According to the Italian guidelines for childhood obesity,^12^ the standard care included a balanced Mediterranean diet using the reference of the Italian Society of Human Nutrition with at least 60 minutes of aerobic activity daily and reduction of sedentary behaviors.

### Outcomes

The primary outcome was the decrease of at least 0.25 BMI SD scores (SDS) at T6. This decrease has been considered the minimal clinically important difference.^12^

The secondary outcomes were changes in waist circumference; serum glucose, insulin, cholesterol, low-density lipoprotein cholesterol (LDL-C), high-density lipoprotein cholesterol (HDL-C), triglyceride, ghrelin, microRNA-221, and interleukin-6 (IL-6) levels; homeostatic model assessment of insulin resistance (HOMA-IR); dietary habits; and GM structure. Lifestyle habits were also monitored.

### Sample Size Calculation

To detect a decrease in BMI SDS greater than or equal to 0.25 in the butyrate arm, we calculated that at least 25 participants per group were necessary at an α level of .05 and a power of 0.90 (Pearson χ^2^ test). Assuming a dropout rate up to 10%, we planned to enroll 27 patients per group.

### Randomization

A central randomization was used to allocate children in a 1:1 ratio into treatment groups. The randomization list was generated applying the *ralloc* command with block sizes of 2 (Stata, version 14.2, StataCorp LLC).

### Allocation Concealment and Blinding

The packaging, color, and weight of the butyrate and placebo capsules were identical. The capsules were provided in sequentially numbered boxes, protected by opaque and sealed envelopes of identical appearance, and prepared by an independent pharmacy according to the randomization list. The investigators, patients, their parents or legal guardians, and the researchers who performed data entry were blinded to group assignment.

### Study Monitoring and Data Management

Study monitoring was performed by an independent clinical trial monitor and included on-site visits and telephone interviews with the investigators. The monitor reviewed the clinical forms for completeness, clarity, and consistency and instructed the researchers to make needed corrections or additions. The clinical researchers (S.C., R.N., C.D.S., A.F.d.G.d.S.S., and R.B.C.) entered data in a case report form. Such data were anonymized and entered into an electronic database by the same researcher. The database underwent data cleaning according to standard procedures and was locked before statistical analysis, performed by a statistician (G.B.).

### Clinical Assessment

At enrollment (T0), the patients’ medical history was collected and recorded in the case report form. At T0 and then monthly (T1-T6), a complete physical examination was performed and data were collected and documented in the case report form.

### Nutritional and Lifestyle Assessment

Anthropometric measurements were performed by the same dietitian (S.C.) at T0 and then monthly until T6. Body weight and height were measured with the children dressed in light indoor clothing and without shoes, using a calibrated mechanical column scale (Astra, GIMA) with an integrated measuring rod. Body mass index was calculated as weight in kilograms divided by height in meters squared, and BMI SDS were obtained from the WHO reference curves. Waist circumference was measured with an anthropometric tape (Seca 203; Seca GmbH) to the nearest 0.1 cm at the midpoint between the iliac crest and the lower rib at the end of a normal expiration. Dietary intake was assessed at T0 and at T6 using a 3-day food record (2 weekdays and 1 weekend day). Parents and tutors of patients received oral and written instructions about how to weigh food and record dietary data. The analysis of energy and nutrient intake was performed using software (Winfood 3; Medimatica Srl). At T6, adherence to the recommendation regarding physical activity and sedentary behaviors was assessed by questioning about lifestyle habits, as previously described.^14^

### Laboratory Assessment

Blood sampling was performed at T0 and T6. Fasting peripheral venous blood samples were taken at 8:30 am and immediately analyzed at the hospital laboratory for glucose, insulin, total cholesterol, HDL-C, LDL-C, and triglyceride levels. HOMA-IR was calculated as glucose level (milligrams per deciliter) × insulin level (microunits per milliliter) / 22.5. Ghrelin and IL-6 levels were measured with commercial enzyme-linked immunoassay kits (Bio-Techne Co and Abcam Inc).

Peripheral blood mononuclear cells were isolated from peripheral whole blood (3 mL) samples using the Ficoll-Paque (GE HealthCare) method. For microRNA expression analysis, total RNA was isolated from peripheral blood mononuclear cells (TRIzol reagent kit; Invitrogen) and quantified (NanoDrop 2000c spectrophotometer; Thermo Scientific), and RNA quality and integrity were assessed (Experion RNA Standard Sense kit, Bio-Rad Laboratories Inc). Quantitative real-time polymerase chain reaction analysis of microRNA-221 was performed (TaqMan miRNA assay kit TaqMan gene expression assay kit, Applied Biosystems). Human peripheral blood mononuclear cells have been previously adopted as surrogates of tissues that are not easily accessible, such as adipose tissue.^15^ MicroRNA-221 was selected for its known association with BMI and with proteins involved in fat metabolism.^16^ Samples were run in duplicate at 95 °C for 15 seconds and at 60 °C for 1 minute (ABI Prism 7900 Sequence Detection System, Applied Biosystems). Data analysis was performed with the comparative threshold cycle method and expressed as 2^−δ^ comparative threshold. MiR-301b was used as reference microRNA for data normalization.

### Metagenome Sequencing and Data Analysis

Stool samples were collected and immediately frozen at −80 °C at T0 and at T6. Twenty-eight samples were insufficient, and sequencing procedures were inadequate for 14 stool samples. Thus, 66 stool samples were analyzed: 36 stool samples from the butyrate arm (n = 18 at T0 and n = 18 at T6) and 30 stool samples from the placebo arm (n = 15 at T0 and n = 15 at T6). Samples were analyzed as previously described.^17,18,19,20,21,22,23,24^ The raw sequence reads have been deposited in the Sequence Read Archive of the National Center for Biotechnology Information under accession number PRJNA788383.

### Adherence and Safety

At each visit, adherence to the allocated treatment was assessed by a count of the number of capsules left in the box. Adherence was defined as the consumption of 100% of the capsules. A dedicated telephone number was available 7 days per week in the case of any suspected adverse event, and at each monthly visit, safety was monitored through a full clinical evaluation. Unscheduled visits were performed if necessary.

### Statistical Analysis

Most continuous variables had nongaussian distributions and are reported as medians (IQRs). Discrete variables are reported as numbers and proportions.

The primary outcome (ie, a decrease of ≥0.25 BMI SDS at T6) was evaluated using a binomial regression model. The response variable of the model was the occurrence of such decrease (discrete: 0 = no, 1 = yes) and the predictor variable was the treatment (discrete: 0 = placebo, 1 = butyrate). The point estimate and 95% CI of the absolute benefit increase (ABI) were obtained from the binomial regression model. The corresponding *P* value was obtained using the Wald test. The 95% CI of the number needed to treat (ie, the number of children needed to treat with butyrate for 6 months to promote the decrease of ≥0.25 of BMI SDS) was calculated using the Bender formula.^13^

We performed an intention-to-treat analysis (ITT) of the primary outcome by considering the children lost to follow-up as all missing values of the primary outcome set to the worst outcome in both butyrate and placebo arms (equal-case scenario ITT) and missing values of the primary outcome set to the worst outcome in the butyrate arm and to the best outcome in the placebo arm (worst-case scenario ITT). The worst outcome was defined as the lack of the decrease of at least 0.25 BMI SDS at T6; the best outcome was defined as the presence of such a decrease at T6. In addition, we performed a per-protocol analysis (PPA) for the primary outcome using only patients who had completed the follow-up period.

The secondary outcomes (ie, changes in waist circumference; fasting glucose, insulin, total cholesterol, LDL-C, HDL-C, triglyceride, microRNA-221, ghrelin, and IL-6 levels; HOMA-IR; dietary and lifestyle habits; and GM structure) were evaluated using PPA. Such changes were evaluated using a linear regression model with the continuous outcome as response variable and treatment (discrete: 0 = placebo, 1 = butyrate), time (discrete: 0 = baseline, 1 = 6 months) and a treatment × time interaction (discrete × discrete) as predictors together with the basal value of the outcome. Cluster 95% CIs were calculated to account for repeated measures. Statistical analysis was performed using Stata, version 17.1 (StataCorp LLC).

Statistical analysis for GM-related outcomes was performed using R, version 4.0.3 (R Foundation for Statistical Computing). Permutational multivariate analysis of variance was performed using R. The R function *pairwise.perm.manova* of the package RVAideMemoire based on 999 permutations was computed on Jaccard distance matrix obtained from MetaPhlAn 3.0 taxonomic profiles. Pairwise Wilcoxon rank sum tests were carried out on taxonomic and functional profiles (function *stat_compare_means* in R package ggplot2). The testing was paired and 2-sided; *P* values <.05 were considered significant.

## Results

The trial was performed from November 1, 2020, to December 31, 2021. A total of 60 children were assessed for eligibility. Five children were excluded because they did not meet inclusion criteria and 1 because of the lack of informed consent. The remaining 54 children (23 boys [43%] and 31 girls [57%]; mean [SD] age, 11 [2.91] years) were randomized in a 1:1 ratio into the butyrate (n = 27) or placebo (n = 27) arm. Four children in the butyrate arm and 2 children in the placebo arm were lost to follow-up, leaving 23 children (85%) in the butyrate arm and 25 children (92%) in the placebo arm (Figure 1).

### Baseline Features

The patients’ baseline features are given in Table 1. The children’s age was higher in the butyrate arm (median [IQR], 12 [9-14] years) than in the placebo arm (median [IQR], 10 [8-12] years). Also, the median SDS of the BMI was higher in the butyrate group (median [IQR], 2.96 [2.54-3.41]) than in the placebo group (median [IQR], 2.80 [2.66-3.53]). In addition to similar IQRs, the distribution of the BMI SDS was similar in the 2 groups (eFigure 1 in Supplement 2). The median of the HOMA-IR was higher in the butyrate vs placebo group (median [IQR], 3.8 [3.0-5.5] vs 2.6 [2.2-3.7]), possibly because of puberty-associated insulin resistance. However, the overall distribution of HOMA-IR in the 2 groups was comparable (eFigure 2 in Supplement 2). The baseline features of patients lost to follow-up are reported in eTable 1 in Supplement 2.

**Table 1.** Baseline Features of the Children

Characteristic	Median (IQR)	
	Placebo (n = 27)	Butyrate (n = 27)
Sex, No. (%)		
Female	13 (48)	18 (67)
Male	14 (52)	9 (33)
Age, y	10 (8-12)	12 (9-14)
Weight, kg	57.6 (50.3-84.0)	68.8 (55.8-79.9)
Height, m	1.46 (1.36-1.56)	1.52 (1.41-1.62)
BMI	27.3 (25.3-31.0)	30.4 (28.3-32.0)
BMI SDS, WHO	2.80 (2.66-3.53)	2.96 (2.54-3.41)
Waist circumference, cm	84.0 (75.5-92.5)	85.0 (79.5-90.0)
Glucose, mg/dL	77 (70-82)	79 (73-87)
Insulin, μU/mL	15 (12-19)	20 (16-28)
HOMA-IR	2.6 (2.2-3.7)	3.8 (3.0-5.5)
Total cholesterol, mg/dL	160 (140-187)	159 (147-194)
LDL-C, mg/dL	104 (83-137)	110 (85-126)
HDL-C, mg/dL	42 (37-54)	43 (38-52)
Triglycerides, mg/dL	86 (55-108)	81 (62-122)
Ghrelin, pg/mL	151 (47-193)	180 (124-224)
microRNA-221, relative expression	4.7 (4.0-5.6)	4.5 (3.6-5.2)
Interleukin-6, pg/mL	6.5 (4.0-9.8)	8.3 (5.0-11.6)
Energy, kcal/d	1747 (1608-2538)	1723 (1485-2403)
Energy, kcal/kg weight	35.2 (23.5-46.6)	26.2 (19.3-39.4)
Carbohydrates, g/d	244 (219-387)	223 (187-342)
Energy from carbohydrates, %	56 (50-63)	53 (49-60)
Fat, g/d	51 (43-69)	57 (36-97)
Energy from fat, %	26 (22-33)	30 (25-37)
SFA, g/d	17 (10-28)	22 (11-34)
Energy from SFA, %	8 (6-10)	10 (7-12)
MUFA, g/d	29 (20-47)	34 (20-53)
Energy from MUFA, %	15 (13-19)	16 (14-20)
PUFA, g/d	6 (5-9)	8 (5-13)
Energy from PUFA, %	3 (3-4)	4 (3-4)
Protein, g/d	79 (66-105)	77 (63-97)
Energy from protein, %	16 (13-20)	17 (13-19)
Protein, g/kg weight	1.4 (1.0-1.9)	1.2 (0.8-1.6)
Cholesterol, g/d	159 (73-236)	186 (163-245)
Fiber, g	16 (11-23)	15 (10-21)
Fiber, g/1000 kcal energy	8 (7-11)	8 (6-11)

Abbreviations: BMI, body mass index (calculated as weight in kilograms divided by height in meters squared); HDL-C, high-density lipoprotein cholesterol; HOMA-IR, homeostatic model assessment of insulin resistance; LDL-C, low-density lipoprotein cholesterol; MUFA, monounsaturated fatty acids; PUFA, polyunsaturated fatty acids; SFA, saturated fatty acids; SDS, SD scores; WHO, World Health Organization.

SI conversion factors: To convert glucose to millimoles per liter, multiply by 0.0555; HDL-C, LDL-C, and total cholesterol to millimoles per liter, multiply by 0.0259; insulin to picomoles per liter, multiply by 6.945; and triglycerides to millimoles per liter, multiply by 0.0113.

### Primary Outcome

The incidence of the primary outcome is reported in Table 2. Assuming an equal-case scenario ITT that all patients lost to follow-up had reached the primary outcome, the ABI was 40% (95% CI, 21%-61%; *P* < .001; n = 54), corresponding to a number needed to treat of 2 (95% CI, 2-5). Under the worst-case scenario ITT (ie, assuming that the 2 patients lost to follow-up in the placebo arm had reached the primary outcome and the 4 patients lost to follow-up in butyrate arm had not), the ABI was 26% (95% CI, 2%-49%; *P* = .03; n = 54) for butyrate vs placebo. In addition, excluding the patients lost to follow-up (ie, performing a PPA), the ABI was 44% (95% CI, 22%-64%; *P* < .001; n = 48) for butyrate vs placebo.

**Table 2.** Incidence of the Main Outcome Decrease in Body Mass Index of at Least 0.25 SDS at 6 Months of Follow-up^a^

Variable	ITT equal-case scenario (n = 54)	ITT worst-case scenario (n = 54)	PPA (n = 48)
Placebo event rate			
No./total No.	15/27	15/27	13/25
Proportion (95% CI)	0.56 (0.37-0.74)	0.56 (0.37-0.74)	0.52 (0.32-0.72)
Butyrate event rate			
No./total No.	26/27	22/27	22/23
Proportion (95% CI)	0.96 (0.89-1.03)	0.81 (0.67-0.96)	0.96 (0.87-1.04)
Absolute benefit increase (butyrate-placebo)^b^			
Proportion (95% CI)	0.40 (0.21-0.61)	0.26 (0.02-0.49)	0.44 (0.22-0.64)
*P* value	<.001	.03	<.001
No. needed to treat, proportion (95% CI)^c^	2 (2-5)	4 (2-88)	2 (2-5)

Abbreviations: ITT, intention-to treat; PPA, per-protocol analysis.

^a^ Values are proportions from binomial regression (see Statistical Analysis for details). The ITT equal-case scenario analysis assumes the occurrence of the best outcome in 4 children lost to follow-up in the butyrate arm and in 2 children lost to follow-up in the placebo arm; the ITT worst-case scenario analysis assumes the occurrence of the worst outcome in 4 children lost to follow-up in the butyrate arm and the best outcome in 2 children lost to follow-up in the placebo arm.

^b^ Wald test used.

^c^ Values were obtained from the Bender formula.

**Figure 2.**
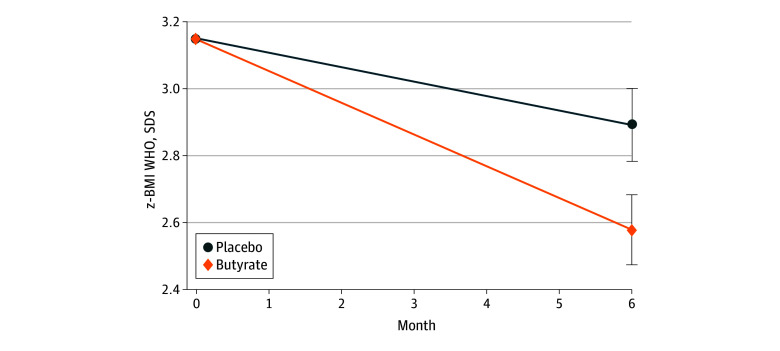
Changes of Body Mass Index (BMI) SD Scores (SDS) in the Placebo and Butyrate Arms The changes of BMI SDS in the placebo and butyrate arms were calculated from linear regression for repeated measures with correction for the basal value of the outcome. WHO indicates World Health Organization.

Figure 2 plots the changes of BMI SDS in the placebo and butyrate arms. The BMI SDS decreased from 3.15 at T0 (95% CI, 3.15-3.16) to 2.89 at T6 (95% CI, 2.78-3.00) in the placebo group and from 3.15 at T0 (95% CI, 3.14-3.16) to 2.58 at T6 (95% CI, 2.48-2.68) in the butyrate group. These changes were calculated from linear regression for repeated measures with correction for the basal value of the outcome (PPA).

### Secondary Outcomes

Table 3 gives the values of anthropometric, laboratory, and dietary data in the placebo and butyrate arms at T0 and at T6 (PPA). Taking into account the baseline values, changes in the outcomes for the butyrate vs placebo group were the following: BMI, −2.26 (95% CI, −3.28 to −1.23; *P* < .001); BMI SDS, −0.31 (95% CI, −0.46 to −0.16; *P* < .001); waist circumference, −5.07 cm (95% CI, −7.68 to −2.46 cm; *P* < .001); insulin level, −5.41 μU/mL (95% CI, −10.49 to −0.34 μU/mL; *P* = .03 [to convert to picomoles per liter, multiply by 6.945]); HOMA-IR, −1.14 (95% CI, −2.13 to −0.15; *P* = .02); ghrelin level, −47.89 μg/mL (95% CI, −91.80 to −3.98 μg/mL; *P* < .001); micro-RNA221 relative expression, −2.17 (95% CI, −3.35 to −0.99; *P* < .001); and IL-6 level, −4.81 pg/mL (95% CI, −7.74 to −1.88 pg/mL; *P* < .001). There were no statistically significant changes in serum glucose, cholesterol, LDL-C, HDL-C, and triglyceride levels.

**Table 3.** PPA of Anthropometric, Laboratory, and Dietary Data in the Placebo and the Butyrate Arms at Baseline and the End of the Trial

Variable	Mean (95% cluster CI)^a^			
	Placebo (n = 25)		Butyrate (n = 23)	
	Baseline	6 mo	Baseline	6 mo
BMI	29.47 (29.35-29.60)	28.71 (27.86-29.56)	29.55 (29.45-29.65)	26.53 (25.94-27.12)
BMI SDS, WHO	3.15 (3.15-3.16)	2.89 (2.78-3.00)	3.15 (3.15-3.16)	2.58 (2.48-2.69)
Waist circumference, cm	86.67 (86.28-87.07)	86.03 (84.94-87.12)	86.87 (86.46-87.28)	81.15 (79.04-83.26)
Glucose, mg/dL	79.45 (78.61-80.28)	74.73 (70.52-78.94)	80.21 (79.48-80.94)	75.43 (69.81-81.05)
Insulin, μU/mL	19.51 (18.28-20.75)	17.16 (14.69-19.64)	20.38 (19.03-21.73)	12.61 (9.3-15.93)
HOMA-IR	3.79 (3.54-4.04)	3.20 (2.73-3.67)	4.00 (3.74-4.27)	2.27 (1.61-2.93)
Total cholesterol, mg/dL	172.39 (167.3-177.49)	160.35 (151.2-169.51)	169.79 (167.73-171.85)	156.75 (149.14-164.35)
LDL-C, mg/dL	115.5 (111.62-119.38)	102.54 (95.21-109.87)	113.08 (111.63-114.53)	99.45 (92.56-106.33)
HDL-C, mg/dL	43.6 (42.85-44.36)	45.19 (41.16-49.23)	43.49 (42.81-44.18)	43.46 (39.36-47.56)
Triglycerides, mg/dL	97.86 (92.31-103.41)	93.14 (79.32-106.96)	98.24 (93.09-103.39)	80.2 (69.47-90.92)
Ghrelin, pg/mL	159.34 (148.15-170.53)	140.94 (120.74-161.15)	164.26 (154.45-174.08)	97.98 (69.24-126.71)
microRNA-221, relative expression	4.42 (4.08-4.77)	3.56 (2.86-4.25)	4.40 (4.14-4.67)	1.36 (0.77-1.95)
Interleukin-6, pg/mL	7.59 (6.88-8.29)	9 (6.79-11.21)	8.15 (7.42-8.88)	4.75 (3.77-5.73)
Energy, kcal/d	2086.85 (1959.19-2214.52)	1595.45 (1380.3-1810.61)	2057.46 (1923.22-2191.7)	1577.07 (1290.38-1863.77)
Energy, kcal/kg weight	33.43 (31.49-35.38)	24.99 (21.48-28.51)	32.12 (29.9-34.35)	27.14 (21.14-33.13)
Carbohydrates, g/d	289.16 (273.24-305.07)	218.63 (177.89-259.38)	282.56 (262.64-302.48)	212.55 (163.67-261.42)
Energy from carbohydrates, %	55.37 (53.78-56.95)	53.89 (48.26-59.51)	53.74 (52.06-55.41)	51.7 (45.8-57.6)
Fat, g/d	66.56 (59.28-73.84)	52.7 (39.51-65.88)	66.94 (60.83-73.06)	54.54 (43.68-65.4)
Energy from fat, %	27.98 (26.93-29.02)	29.27 (24.87-33.67)	29.11 (27.72-30.5)	32.21 (28.09-36.33)
Energy from SFA, %	8.84 (8.23-9.45)	7.72 (6.1-9.34)	9.19 (8.63-9.75)	8.46 (7.35-9.56)
Energy from MUFA, %	15.6 (14.98-16.22)	17.46 (15.00-19.92)	16.21 (15.38-17.05)	19.43 (16.63-22.23)
Energy from PUFA, %	3.46 (3.15-3.76)	4.02 (3.13-4.91)	3.79 (3.39-4.19)	4.4 (3.56-5.24)
Protein, g/d	82.55 (77.12-87.98)	61.42 (52.21-70.63)	81.44 (76.49-86.4)	59.27 (48.65-69.9)
Energy from PRO, %	16.77 (15.95-17.59)	16.96 (14.15-19.77)	17.03 (16.13-17.94)	15.97 (13.43-18.5)
Protein, g/kg weight	1.35 (1.25-1.45)	0.96 (0.80-1.11)	1.28 (1.18-1.37)	0.99 (0.8-1.18)
Cholesterol, g/d	196.11 (168.24-223.98)	106.25 (60.18-152.32)	213.9 (185.73-242.07)	143.09 (92.5-193.69)
Fiber, g	17.72 (15.85-19.6)	17.99 (13.01-22.96)	16.94 (15.27-18.61)	15.06 (10.75-19.36)
Fiber, g/1000 kcal energy	8.57 (7.93-9.21)	11.45 (8.88-14.03)	8.51 (7.75-9.28)	9.14 (7.67-10.61)

Abbreviations: BMI, body mass index (calculated as weight in kilograms divided by height in meters squared); HDL-C, high-density lipoprotein cholesterol; HOMA-IR, homeostasis model assessment of insulin resistance; LDL-C, low density lipoprotein-cholesterol; MUFA, monounsaturated fatty acids; PPA, per protocol analysis; PUFA, polyunsaturated fatty acids; SDS, SD scores; SFA, saturated fatty acids; WHO, World Health Organization.

SI conversion factors: To convert glucose to millimoles per liter, multiply by 0.0555; HDL-C, LDL-C, and total cholesterol to millimoles per liter, multiply by 0.0259; insulin to picomoles per liter, multiply by 6.945; and triglycerides to millimoles per liter, multiply by 0.0113.

^a^ Values were obtained from linear regression for repeated measures with correction for the basal value of the outcome. Details are presented in the Statistical Analysis section.

Changes in the diet energy and nutrient content were similar across arms, suggesting a comparable pattern of adherence to dietary advice (Table 3). In particular, at T6, a reduction of daily energy intake and a macronutrient redistribution toward the recommended range and the Mediterranean diet principles were observed in both groups. Furthermore, a similar pattern of adherence to lifestyle recommendations was observed at T6 in the 2 groups for all the physical activity and sedentary behaviors scores (eTable 2 in Supplement 2).

We did not find significant between-arm differences in the GM taxonomic structure, both at T0 and at T6 (permutational multivariate analysis of variance). However, we detected an association of the baseline GM structure with the response to the intervention. In particular, the baseline abundance of *Faecalibacterium prausnitzii* was associated with the decrease in HOMA-IR and that of *Roseburia faecis* was associated with a decrease in insulin levels, while *Ruminococcus torques* showed a negative correlation with HOMA-IR (eFigure 3 in Supplement 2). In addition, patients receiving butyrate showed an increase in gene richness and a decrease in genes involved in branched-chain amino acids biosynthesis, as determined by the Wilcoxon rank sum test.

### Adherence

The study procedures and intervention were well accepted by patients. Adherence to the treatment was lower in the butyrate vs placebo group, with 4 vs 2 children refusing to continue treatment. Adherence evaluation revealed that these 4 patients stopped butyrate consumption after their first doses.

### Adverse Effects

No patients reported adverse effects in the placebo arm. Transient mild nausea and headache were reported by 2 patients during the first month of butyrate intervention. Both symptoms disappeared during the following 4 weeks, and both patients were able to continue the assigned treatment. No drug therapy was required for the treatment of these symptoms.

## Discussion

Emerging data suggest that butyrate is a health-promoting molecule able to modulate energy homeostasis, insulin sensitivity, lipid metabolism, and inflammation.^8^ A deficiency of butyrate has been linked to deleterious effects on human metabolism^25^ and has been reported in individuals with obesity.^26^

To our knowledge, the BAPO trial was the first randomized clinical trial evaluating the effects of butyrate on pediatric obesity and showed that a 6-month supplementation can reduce BMI and ameliorate glucose metabolism and inflammation. The beneficial effects of butyrate administration on glucose metabolism were consistent with previous data.^27^ Butyrate supplementation improved insulin sensitivity, limiting insulin resistance and hyperinsulinemia in animals^28,29^ and in lean humans.^30^ Butyrate supplementation decreased HOMA-IR levels in adults with diabetes.^31^ In line with these data,^27,28,29,30,31^ we found that butyrate supplementation decreased HOMA-IR and fasting insulin levels in children with obesity. Furthermore, the GM analysis supported the role of butyrate in glucose metabolism, as suggested by a more positive response in children with a higher abundance of butyrate-producing bacteria at baseline.

Supplementation with inulin, a dietary substrate for butyrate production, determined reduction of appetite, body weight, and of the orexigenic hormone ghrelin in rats.^32^ Indeed, butyrate is involved in the gut-brain axis through the modulation of hormones regulating satiety and appetite signaling.^33,34,35^ In keeping with these findings, we found that butyrate supplementation elicited a significant reduction of ghrelin serum levels in children with obesity.

Abdominal obesity is associated with negative health outcomes.^36^ Waist circumference is a marker of abdominal obesity.^37^ In line with previous data, we found that butyrate produced a significant decrease in waist circumference in children with obesity, likely owing to its lipolytic action.^38,39^

MicroRNAs are involved in adipogenesis and have metabolic and endocrine functions.^40^ Previous studies showed that microRNA-221 was upregulated in patients with obesity with increased insulin resistance, BMI, and inflammation.^16,41,42^ We found that butyrate reduced microRNA-221 expression, and the effect paralleled with BMI, HOMA-IR, insulin, and the proinflammatory cytokine IL-6 level reduction. A similar modulatory action on IL-6, the major circulating inflammatory mediator in individuals with obesity,^43^ has been reported in preclinical studies.^44,45^

### Strengths and Limitations

To our knowledge, the BAPO trial was the first randomized clinical trial evaluating the effects of butyrate supplementation in children with obesity on BMI and several variables involved in the pathogenesis of obesity. This trial has limitations. The main limitations are the lack of data regarding body composition, resting energy expenditure, other metabolic variables, and butyrate serum levels as objective markers of intervention adherence. In addition, the lack of use of monitor-based devices that objectively quantify movement could also be considered a limitation of the study.

## Conclusions

The findings of this study suggest that in children with obesity, oral butyrate supplementation may produce a reduction of BMI and exerts beneficial effects on glucose metabolism and inflammation. These data support the importance of the GM-derived metabolite butyrate as a protective factor against obesity, highlighting the central role of a healthy diet and GM function to achieve an optimal endogenous production of butyrate. Randomized clinical trials with longer follow-up are needed to confirm our findings.

The lower adherence rate and higher frequency of mild adverse effects observed in patients treated with butyrate suggest that the unpleasant organoleptic features of this compound may limit its clinical application. Hence, development of new butyrate-based compounds free of these unpleasant features is advocated for a more effective therapeutic action of butyrate against pediatric obesity.

## Notes

Supplement 1.
Trial Protocol

Supplement 2. **eFigure 1.** The Distribution of BMI (SDS WHO) at Baseline **eFigure 2.** The Distribution of HOMA-IR (Unitless) at Baseline **eFigure 3.** GM Signatures at Baseline Predictable of the Metabolic Response to the Intervention **eTable 1.** The Baseline Features of Patients Lost to Follow-up **eTable 2.** The Physical and Sedentary Behaviors Questionnaires Results

Supplement 3.
Data Sharing Statement
