# Error in Funding/Support

**DOI:** 10.1001/jamanetworkopen.2024.21429

**Published:** 2024-06-06

In the Original Investigation titled “Therapeutic Effects of Butyrate on Pediatric Obesity: A Randomized Clinical Trial,”^1^ published December 5, 2022, the Funding/Support section was updated to include additional information on the National Recovery and Resilience Plan project that supported the study. This article has been corrected.^1^
