# Error in Funding/Support

**DOI:** 10.1001/jamanetworkopen.2025.9711

**Published:** 2025-04-08

In the Original Investigation titled “Therapeutic Effects of Butyrate on Pediatric Obesity: A Randomized Clinical Trial,”^1^ published December 5, 2022, the Funding/Support section was updated to remove the entity “the Department of Translational Medical Science of the University of Naples” and replace the funding number D93C22000890001 with E63C22002030007. This article has been corrected.^1^ This article was previously corrected on June 6, 2024, to fix earlier errors in Funding/Support.^2^
