# Supplementary material for: Therapeutic Effects of Butyrate on Pediatric Obesity: A Randomized Clinical Trial
Source: JAMA Netw Open. 2022 Dec 5;5(12):e2244912. doi: 10.1001/jamanetworkopen.2022.44912 (PMC9855301; doi:10.1001/jamanetworkopen.2022.44912)
Supplement: Supplement 1. — Trial Protocol [file jamanetwopen-e2244912-s001.pdf]

University of Naples “Federico II” - Naples, Italy

STUDY PROTOCOL

Title

Therapeutic Effects of Butyrate Against Pediatric Obesity (BAPO)

Principal Investigator

Roberto Berni Canani, MD, PhD

Department of Pediatrics, University of Naples “Federico II”, Naples, Italy

E mail: berni@unina.it

Type of research

Quadruple-Blind Randomized, Monocentric, Placebo-controlled Study. No profit.

University of Naples “Federico II” - Naples, Italy

Background

Childhood obesity has emerged as an important global health concern that is associated with emergence of comorbidities previously considered to be “adult” diseases including type 2 diabetes mellitus, hypertension, nonalcoholic fatty liver disease, obstructive sleep apnea, and dyslipidemia (1).

According to the World epidemiological data of WHO 38 million children under 5 years were overweight or obese in 2019 and over 340 million children and adolescents aged 5-19 were overweight or obese in 2016 (2).

Obesity arises from complex interactions between genes and environmental factors such as diet, food components and/or way of life, and results from a long-term positive imbalance between energy intake and expenditure with excessive increase in body fat (3).

It is now well established that gut microbiota (GM) can influence human health and a growing body of evidence suggests that GM play a metabolic role in energy regulation and substrate metabolism (4). Various factors can impact GM and several underlying mechanisms have been proposed, one of these are fermentable dietary fibers that represent the fraction of not digested food by endogenous enzymes in the small intestine which are converted into an array of small organic metabolites by microbes in the large intestine, the most important are short-chain fatty acids (SCFA)

acetate, propionate and butyrate (5, 6). Of the SCFAs, in particular butyrate supplementation was found to have multiple metabolic benefits in murine models, including prevention of high-fat diet (HFD) induced obesity, insulin resistance (IR) and hepatic steatosis (7, 8, 9,10).

With the dramatically increase of obesity prevalence seen in the pediatric population, novel insights are necessary to counteract this epidemic disease, so a reasonable speculation is that butyrate acts on components of the energy balance, that is, stimulating energy expenditure, and/or reducing energy intake, thereby reducing obesity and obesity-associated disorders.

Li et al (11) report that acute oral butyrate administration decreased food intake in HFD fed mice, suppressed the activity of orexigenic NPY neuron activity in the hypothalamus indicating the effect of butyrate on satiety; in addition to inducing satiety, butyrate also promoted fat oxidation and activated brown adipose tissue, evident from increased utilization of plasma triglyceride-derived fatty acids.

Alternatively, the metabolic benefits of butyrate may be induced by satiety gut hormone Peptide YY (12) or by stimulation of GLP-1 secretion from L cells of the gastrointestinal (GI) tract, which activates GLP-1 receptor signalling in the vagal nerve and consequently induces hypothalamic satiety signaling (13, 14).

In this context oral butyrate administration in obese children could induce body weight loss, that can be achieved by decreasing energy intake, reducing the consumption or absorption of food, and/or by increasing energy expenditure.

The aim of this study is to evaluate the effects of 6-month oral butyrate supplementation compared to placebo in a cohort of obese pediatric patients in addition to standard care for pediatric obesity treatment.

References

1. Kumar S., Kelly A.S. Review of Childhood Obesity: From Epidemiology, Etiology, and Comorbidities to Clinical Assessment and Treatment. Mayo Clin Proc. 2017 Feb;92(2):251-265.

2. World Health Organization. Obesity and overweight. Fact sheet no 311 January 2015. [cited 2016 20 April 2016; Available from] http://www.who.int/mediacentre/factsheets/fs311/en/.

3. Gérard P. Gut Microbiota and Obesity. Cell Mol Life Sci. 2016 Jan;73(1):147-62.

4. Bauer P.V., Hamr S.C., Duca F.A. Regulation of Energy Balance by a Gut-Brain Axis and Involvement of the Gut Microbiota. Cell Mol Life Sci. 2016 Feb;73(4):737-55.

5. Canfora E.E., Jocken, J.W. Blaak, E.E. Short-chain fatty acids in control of body weight and insulin sensitivity. Nat Rev Endocrinol. 2015 Oct;11(10):577-91.

6. Knudsen K.E.B., Lærke H.N., Hedemann M.S., Nielsen T.S., Ingerslev A.K., Nielsen D.S.G., Theil P.K., Purup S., Hald S., Schioldan A.G., Marco M.L., Gregersen S., Hermansen K. Impact of Diet-Modulated Butyrate Production on Intestinal Barrier Function and Inflammation. Nutrients. 2018 Oct 13;10(10):1499.

7. Khan S., Jena G. Sodium butyrate reduces insulin-resistance, fat accumulation and dyslipidemia in type-2 diabetic rat: a comparative study with metformin. Chem Biol Interact. 2016 Jul 25;254:124-34.

8. Gao Z., Yin J., Zhang J., Ward R.E., Martin R.J., Lefevre M., Cefalu W.T., Ye J. Butyrate improves insulin sensitivity and increases energy expenditure in mice. Diabetes. 2009 Jul;58(7):1509-17.

9. Henagan T.M., Stefanska B., Fang Z., Navard A.M., Ye J., Lenard N.R., Devarshi P.P. Sodium butyrate epigenetically modulates high-fat diet-induced skeletal muscle mitochondrial adaptation, obesity and insulin resistance through nucleosome positioning. Br J Pharmacol. 2015 Jun;172(11):2782-98.

10. Mattace Raso G., Simeoli R., Russo R., Iacono A., Santoro A., Paciello O., Ferrante M.C., Berni Canani R., Calignano A., Meli R.

Effects of sodium butyrate and its synthetic amide derivative on liver inflammation and glucose tolerance in an animal model of steatosis induced by high fat diet. PLoS One. 2013 Jul 5;8(7):e68626.

11. Li Z., Yi C.X., Katiraei S., Kooijman S., Zhou E., Chung C.K., Gao Y., van den Heuvel J.K., Meijer O.C., Berbée J.F.P., Heijink M., Giera M., van Dijk K.W., Groen A.K., Rensen P.C.N., Wang Y. Butyrate reduces appetite and activates brown adipose tissue via the gut-brain neural circuit. Gut. 2018 Jul;67(7):1269-1279.

12. Brooks L., Viardot A., Tsakmaki A., Stolarczyk E., Howard J.K., Cani P.D., Everard A., Sleeth M.L., Psichas A., Anastasovskaj J., Bell J.D., Bell-Anderson K., Mackay C.R., Ghatei M.A., Bloom S.R., Frost G., Bewick G.A. Fermentable carbohydrate stimulates FFAR2-dependent colonic PYY cell expansion to increase satiety. Mol Metab. 2016 Nov 4;6(1):48-60.

13. Lin H.V., Frassetto A., Kowalik E.J., Nawrocki A.R., Lu M.M., Kosinski J.R., Hubert J.A., Szeto D., Yao X., Forrest G., Marsh D.J. Butyrate and propionate protect against dietinduced obesity and regulate gut hormones via free fatty acid receptor 3-independent mechanisms. PLoS One. 2012;7(4):e35240.

14. Tolhurst G., Heffron .H, Lam Y.S., Parker H.E., Habib A.M., Diakogiannaki E., Cameron J., Grosse J., Reimann F., Gribble F.M.

Short-chain fatty acids stimulate glucagonlike peptide-1 secretion via the g-protein-coupled receptor FFar2. Diabetes. 2012 Feb;61(2):364-71.

Aims

Main study outcome

- To determine the effectiveness on child weight status (BMI z-score reduction)

Secondary study outcomes

- To evaluate glucose metabolism parameters (glycemia, insulinemia, HOMA Index), lipid metabolism parameters (total cholesterol, LDL, HDL and triglycerides), serum levels of the hormone ghrelin and of the proinflammatory cytokine IL-6, and on the expression of microRNA-221;
- To assess dietary and lifestyle habits;
- To assess metagenomic characteristic of the gut microbiota.

Population

Inclusion criteria

Children/adolescents with diagnosis of obesity (BMI >95° percentile for age and sex), 5-17 years, observed at Pediatric Section of Department of Translational Medical Sciences (University of Naples Federico II).

Exclusion criteria

- Age at enrollment <5 or >17 years
- BMI <95° percentile for age and sex
- Patients under pharmacological treatment for obesity (metformin)
- Patients assuming vitamin E,
- Patients assuming pre-, pro- or synbiotics
- Simultaneous presence of other chronic diseases unrelated to obesity: cancer, immunodeficiency, cystic fibrosis, allergies, celiac disease, autoimmune diseases, neuropsychiatric disorders, type 1 diabetes, inflammatory bowel diseases, malformations of urinary or gastrointestinal or respiratory tract, chronic lung diseases, genetic and metabolic diseases, chronic hematological diseases.
- History of surgery for the treatment of obesity
- Any medical condition that may interfere with participation in this study
- Participation in other clinical trials still in progress

Study Design

Quadruple-Blind Randomized, Monocentric, Placebo-controlled Study.

Obese patients related to the Pediatric Section of Department of Traslational Medical Science of the University of Naples "Federico II" will be evaluated for the study.

At the baseline, inclusion and exclusion criteria will be assessed and the aims and methods of the study will be illustrated to parents/tutors and young patients. After obtaining informed consent from the patients and their parents/tutor (Annex 1.), patients will be enrolled and randomly assigned to two group of 6-month intervention.

Group 1: standard care for pediatric obesity + sodium butyrate (20 mg/kg body weight/day)

Group 2: standard care for pediatric obesity + placebo (cornstarch)

The packaging, color, weight, smell and taste of the active product and of the placebo will be identical without indication of group identity or content and will labeled with a serial number according to the computer-generated randomization list, and thus ensured blind conditions.

Instructions for keeping and maintaining the product will be also providing.

All study subjects will be evaluated by a multidisciplinary team (composed by pediatricians, nutritionists and pediatric nurses experienced in the care of pediatric obesity).

Anamnestic, demographic, anthropometric and clinical data will be

collected and reported in a specific clinical chart (Annex 2.).

The patients and their parents/tutor will be instructed on how to complete a food record (Annex 3.) for the evaluation of dietary patterns which will subsequently be analyzed by a specific software (Winfood - Medimatica Srl). Compliance with the recommendation regarding physical activity and sedentary behaviors at T6 was assessed by questioning about lifestyle habits (Annex 4.).

At enrollment (T0) and every month for 6 months (T1, T2, T3, T4, T5, T6) will be evaluated: body weight, height, BMI, weight for age percentile, height for age percentile, BMI for age percentile, waist circumference, blood pressure, eating behavior (3 days food record).

At the baseline (T0) and at the end of the study (T6) peripheral blood sampling will be performed for the evaluation of fasting blood glucose, basal insulin, HOMA-IR index, triglyceride, HDL cholesterol, LDL cholesterol, total cholesterol, ghrelin, and expression profile from PBMCs of miR-221. At enrollment (T0), before the start of interventions, and after 6 months (T6) a stool sample for each patient (3 g) will be collected for the metagenomic analyses of the gut microbiota. All samples will be collected from diapers in sterile plastic tubes and stored at -80°C until analysis.

Laboratory determinations

Blood sampling will be performed at recruitment (T0) and at the end of the study (T6). Fasting peripheral venous blood samples will be taken at 8:30

a.m. and immediately analyzed at the hospital laboratory of biochemistry for total cholesterol, HDL-cholesterol, LDL-cholesterol, triglycerides, insulin, and glucose. The HOMA-IR will be calculated as: the product of fasting glucose (mg/dl) and fasting insulin (µUI/ml) divided by 22.5.

To detect serum levels of ghrelin and IL-6 specific Human Ghrelin ELISA Kit (Biotechne, Minneapolis, USA) and IL-6 Human ELISA kit (Abcam, Cambridge, UK) will be used, with a detection limit of 9.38 pg/mL and 6.25 pg/ml, respectively.

Peripheral blood mononuclear cells (PBMCs) will be isolated from peripheral whole-blood (3ml) samples using the Ficoll-Paque (GE Health-care, Uppsala, Sweden) method. For microRNA (miRNA) expression analysis, total RNA will be isolated from PBMCs using the TRIzol Reagent kit (Invitrogen, Carlsbad, CA, USA) and quantified with the NanoDrop 2000c spectrophotometer (Thermo Scientific, Waltham, MA, USA). RNA quality and integrity will be assessed with the Experion RNA Standard Sense kit (Bio-Rad, Hercules, CA, USA). Quantitative real-time PCR analysis of the miR-221 will be performed with the TaqMan miRNA assay kit and the TaqMan gene expression assay kit, respectively (both from Applied Biosystems,Grand Island, NY, USA) according to the manufacturer’s instructions. Samples will run in duplicate at 95°C for 15 seconds and 60°C for 1 minute using an ABI Prismâ 7900 Sequence Detection System (Applied Biosystems). Data analysis will be performed with the comparative threshold cycle (CT) method and expressed as 2^-

delta CT. MiR-301b will be used as reference miRNA for data normalization.

Stool samples will be collected and immediately frozen at −80°C at enrolment (T0) and at the end of the study (T6). DNA extraction from fecal samples will be carried out following the SOP 07 developed by the International Human Microbiome Standard Consortium (www.microbiome-standards.org). DNA libraries will be sequenced on Illumina NovaSeq platform, leading to 2x150bp, paired-end reads.

Compliance and safety monitoring

At each visit, the compliance to the allocated treatment will be assessed based on number of capsules unit left in the box. Compliance to the allocated treatment was defined as the consumption of 100% of the capsules. Furthermore, compliance with the recommendation regarding physical activity will be assessed by questions about daily exercises.

A dedicated phone number was available 7 days per week for parents/legal guardians of each patient in the case of occurrence of any suspected adverse event and at each monthly visit the safety will be monitored through a full clinical evaluation. Unscheduled visits will be performed if necessary.

Sample size

Considering a possible drop-out of 10%, will be required 27 patients per group (Group 1: n=27; Group 2: n=27) to observe a BMI z-score reduction at least of ≥0.25 after 6 months of intervention (Power of the study 90%; type 1 error =0.050, 2- tailed test).

Statistical analysis

The Kolmogorov-Smirnov test will be used to determine whether variables will be normally distributed. For continuous variables the study groups will be compared using the mean equality test or, if necessary, the Mann-Whitney U test. The Kruskal-Wallis H-test will be used to compare the two groups. The comparison between two groups and between two samples will be performed using Welch's t test and Fisher's exact test respectively (with Bonferroni's correction). The $\chi 2$ test and Fisher’s exact test will be used for categorical variables. The data obtained will be assessed by means of linear logistic regression analysis in order to evaluate the possible influence of dietary factors and other variables (anthropometric data, glucose and lipid metabolism variables). We will use a generalized linear regression model (GLM) to examine the contribution of demographic, anamnestic and clinical data of children enrolled on the composition of the microbiota. A GLM model will be built and validated using the rms and ResourceSelection packages implemented in R respectively (http: // www.r-project.org/). Multivariate statistical analysis (Analysis of the Main

Components, hierarchical clustering, Analysis of the Main Coordinates, etc.) will be used to identify possible groupings of the samples and the variables that allow to explain these subdivisions. All statistical tests will be carried out in the R / Bioconductor environment and using specific software (stats, vegan, made4, rgl, etc.). The relative abundance tables of the microbial species will also be used for correlation analysis with the other quantitative variables available using the psych software in the R environment. The level of significance for all statistical tests will be 2-sided, $p<0.05$. When appropriate, all significance values will be corrected to take into account the effect of multiple comparisons, using the Bonferroni correction. All data were collected in a dedicated database and analyzed by a statistician blinded to patient group assignment, using SPSS version 19.0 for Windows (SPSS Inc, version 14.0, Chicago, IL).

Timeline

- 0-3 months: patients recrutiments and enrollment
- 3-9 months: first follow-up of patients treated with butyrate or placebo
- 9-12 months: data analysis and preparation of a first draft of the results

University of Naples “Federico II” - Naples, Italy

Annex 1.

Informative Model

Therapeutic Effects of Butyrate Against Pediatric Obesity (BAPO)

Childhood obesity has emerged as an important global health concern, that is associated with severe comorbidities including type 2 diabetes mellitus, hypertension, nonalcoholic fatty liver disease, obstructive sleep apnea, and dyslipidemia. According to the World epidemiological data of WHO 38 million children under 5 years were overweight or obese in 2019 and over 340 million children and adolescents aged 5-19 were overweight or obese in 2016. Obesity arises from complex interactions between genes and environmental factors such as diet, food components and/or way of life, and results from a long-term positive imbalance between energy intake and expenditure with excessive increase in body fat. It is now well established that gut microbiota (GM) can influence human health and a growing body of evidence suggests that GM play a metabolic role in energy regulation and substrate metabolism. Various factors can impact GM, one of these are fermentable dietary fibers that represent the fraction of not digested food by endogenous enzymes in the small intestine which are converted into an array of small organic metabolites by microbes in the large intestine, the most important are short-chain fatty acids (SCFA) acetate, propionate and butyrate. In particular, it has shown that butyrate exerts multiple metabolic

benefits in murine models, including prevention of obesity, insulin resistance and hepatic steatosis.

With the dramatically increase of obesity prevalence seen in the pediatric population, novel insights are necessary to counteract this epidemic disease, so a reasonable speculation is that butyrate acts on components of the energy balance, that is, stimulating energy expenditure, and/or reducing energy intake, thereby reducing obesity and obesity-associated disorders.

The aim of this project is to determine the effectiveness of 6-month oral butyrate supplementation on child weight status (BMI z-score reduction), compared to placebo, in a cohort of pediatric obese patients in addition to standard care for pediatric obesity treatment.

We request your adherence to this research project which, if successful, thanks to your collaboration, will help us to collect useful data to better manage and counteract pediatric obesity.

Children participating in the study will be randomly assigned to two group of 6-month intervention: one group will receive an oral administration of of sodium butyrate and the other group will receive an oral administration of placebo, both groups will also receive standard lifestyle indication for obesity treatment. During the first visit and every month for 6 months will be evaluated clinical and anthropometric parameters and dietary habits of all participating children. At first visit and at the end of the study will be performed a peripheral blood sampling, indirect calorimetry and a stool

sample for each patient (3 g) will be collected for gut microbiota analyses. Any decision to not participate or to stop participating in the study may occur at any time even without providing any explanation or justification, will not imply any penalty, loss of benefits or loss of legal rights for you and your child. Any information collected during the study and in particular personal data will be considered strictly confidential and will be kept in an absolutely anonymous manner, so as not to make any identifiable the identity of the recruited person. The decision to participate in this study is completely voluntary. Participation in the study will not expose the child to any risk. The doctor in charge of the study will always be at your disposal for any clarification. The study will involve 54 children and it will last for 12 months.

The doctor in charge of the study (Prof. Roberto Berni Canani, Department of Translational Medical Sciences of the "Federico II" University of Naples) will always be at your disposal every weekday from 9 to 17:30 for any clarification (e- mail: berni@unina.it; Telephone / Fax: + 39 081 746 2680). The study was approved by the Ethics Committee of University of Naples “Federico II”, Naples, Italy.

University of Naples “Federico II” - Naples, Italy

Informed consent of the parent/caregiver

Parent/caregiver

I have received from Dr. .... the proposal to participate in the study called "*Therapeutic Effects of Butyrate Against Pediatric Obesity (BAPO)*" approved by the Ethics Committee of University of Naples “Federico II”, Naples, Italy. I have received the information document describing the study. The study and the conditions for participation have been adequately explained to me. I have read the information document. I have had the opportunity to ask questions. I am aware of the fact that I am not obliged to have my child participate in this study and to be able to withdraw it at any time without giving any justification and without any disadvantage. I consent to participate in this scientific study

Name………………. Surname…………………………

Parent / caregiver of the minor ....

Signature ....

Place and date ....

Doctor

I have obtained the above consent. I confirm that I have provided the minor and his / her parents / caregiver with exhaustive explanations about the nature, purpose, duration and possible risks associated with the

experimentation in question, which I believe have been understood in conscience.

Investigator.

Name ……………..Surname……..………………….

Signature............................. ..

Place and date ....

Below is the information and model of the consent to the processing of personal data according to the "Guidelines for the processing of personal data in the context of clinical trials of medicines" - 24 July 2008 - G.U. n. 190 of August 14, 2008.

Information and manifestation of the consent of the parent / caregiver to the processing of personal data (1)

Data controllers and related purposes The Experimental Center, Federico II University of Naples, which commissioned the study that was described to you, within the scope of its competence and in accordance with the responsibilities established by the rules of good clinical practice (legislative decree 211/2003), will process the personal data of your child, in particular those on health, only insofar as they are indispensable in relation to the objective of the study, exclusively according to the

realization of the study. To this end, the data indicated will be collected by the Principal Investigator Prof. Roberto Berni Canani (2). The processing of personal data, especially those on health is essential to the conduct of the study: the refusal to give them will not allow your child to participate.

Nature of data

The doctor who will follow your child in the study will identify him / her with a code: the data concerning him / her collected during the course of the study, with the exception of the name, will be recorded, processed and stored together with code. Only the doctor and authorized persons can link this code to your child's name.

Processing methods

The data, processed by electronic means, will be disseminated only in strictly anonymous form, for example through scientific publications, statistics and scientific meetings. The participation of your child / child at the study implies that, in accordance with the legislation on clinical trials of medicines, the Ethics Committee and the Italian and foreign health authorities will be able to know the data concerning your child, also contained in the original clinical documentation, in such a way as to guarantee the confidentiality of the identity.

Exercise of rights

You can exercise the rights referred to in art. 7 of the Code (eg access to personal data of your child, integrate them, update them, rectify them, oppose their treatment for legitimate reasons, etc.) by contacting the

Principal Investigator directly (Prof. R. Berni Canani-Department of Translational Medical Sciences-University of Naples Federico II Via S. Pansini 5, 80131 Naples). You may interrupt your child's participation in the study at any time without providing any justification: in this case, the biological samples related to your child will be destroyed. Further data concerning your child will not be collected, without prejudice to the use of those already collected to determine, without altering, the results of the research.

Consent

I consent to the processing of my child's personal data, by signing this form, for the purposes of the research within the limits and with the methods indicated in the information provided with this document.

Name and surname of the interested party (in block letters)

____

Parent/caregiver of

____

Signature of the interested party ________________________

Data ________________________

(1) To be submitted to the interested parties together with the informed consent form that describes the scientific characteristics of the study, including by integrating it. (2) When it is not possible to know at the time of drafting the information the complete list of third parties to which data will be transmitted also in non-EU countries it is necessary to specify how

and when the complete list will be made available.

Respect for privacy

Respecting privacy, the researchers involved in the study will keep the medical and clinical data collected regarding the subject and the family in such a way as to guarantee absolute anonymity.

Disclosure of the results obtained

The information obtained as results of the study will be disseminated to the participants within 3 months from the end of the study.

Contacts

Roberto Berni Canani, MD, PhD

Department of Translational Medical Sciences - Section of Pediatrics and European Laboratory for the Study of Food-induced Diseases, University of Naples "Federico II", Naples

Via Pansini 5, 80131, Naples, Italy

e-mail: berni@unina.it

Telephone / Fax: + 39 081 746 2680 (every weekday from 9 a.m to 5:30 p.m).

University of Naples “Federico II” - Naples, Italy

Annex 2.

Anamnestic, demographic, anthropometric and clinical data

First visit: …/…/…<br>***Patient ID:***<br>Name and Surname:<br>Place and birth date:	
Telephone Number:	
**Inclusion Criteria**	
• Caucasian children, both sexes	☐
• Aged 5-17 years	☐
• Diagnosis of obesity (Body Mass Index at or greater than 95th percentile for age and sex)	☐
**<u>Exclusion Criteria</u>**	
- Age at enrollment <5 or >17 years	☐
- BMI <95° percentile for age and sex	☐
- Patients under pharmacological treatment for obesity (metformin)	☐
- Patients assuming vitamin E,	☐
- Patients assuming pre-, pro- or synbiotics	☐
- Simultaneous presence of other chronic diseases unrelated to obesity: cancer, immunodeficiency, cystic fibrosis, allergies, celiac disease, autoimmune diseases,	☐

neuropsychiatric disorders, type 1 diabetes, inflammatory bowel diseases, malformations of urinary or gastrointestinal or respiratory tract, chronic lung diseases, genetic and metabolic diseases, chronic hematological diseases.	
- History of surgery for the treatment of obesity	☐
- Any medical condition that may interfere with participation in this study	☐
- Participation in other clinical trials still in progress	☐
**Enrollment** *(for the presence of all the inclusion criteria and the absence of all the exclusion criteria)*<br>Yes ☐ No ☐	
**Group**<br>1 ☐ 2 ☐	

	Enrollment	V1	V2	V3	V4	V5	V6
**Body Weight**							
**Height**							
**BMI**							
**Weight for age percentile**							
**Height for age percentile**							
**BMI for age percentile**							
**Waist**							

**circumference**							
**Blood pressure**							
**Food record**	Yes ☐<br>No ☐	Yes ☐<br>No ☐	Yes ☐<br>No ☐	Yes ☐<br>No ☐	Yes ☐<br>No ☐	Yes ☐<br>No ☐	Yes ☐<br>No ☐
**Fasting blood glucose**							
**Basal insulin**							
**HOMA-index**							
**Triglycerides**							
**HDL cholesterol**							
**LDL cholesterol**							
**Total cholesterol**							
**Ghrelin**							
**IL-6**							
**Expression profile of miR-221**							
**Stool sample**	Yes ☐<br>No ☐						Yes ☐<br>No ☐

Annex 3.

Food record

INSTRUCTIONS FOR THE COMPLILATION:

1. Report all foods (including: snacks, candies, gums, etc ...) and all drinks (including tea, chamomile, water) that you consume throughout the day. Report the drugs, supplements and vitamin and mineral supplements on the page at the bottom of the diary.
2. Carefully report all the information in the appropriate boxes, writing in block letters.
3. As a quantity, report only what you really consumed.
4. In the case of cooked dishes, indicate the exact name of the recipe and / or the type of cooking and, if you know them, the ingredients.
5. When you consume packaged food, look on the label for the exact brand and name of the product to accurately report it on the card.
6. Always specify in detail the food consumed, for example milk (skimmed, partially skimmed, whole, etc ...), meat (beef, turkey, chicken, etc ...), oil (olive, extra virgin olive oil olive, seeds, etc ...).
7. Remember to put all the condiment you added in the appropriate column (parmesan, ketchup, oil, sugar, butter, etc ...).

University of Naples “Federico II” - Naples, Italy

Food record Surname:…………………Name:…………………

Date: …/…/…

Visit:

1 ☐ 2 ☐ 3 ☐ 4 ☐ 5 ☐ 6 ☐

	**Time and place**	**Food and Drinks** *(Write only one food per box, in the case of recipes or packaged foods, list the ingredients or indicate the brand)*		**CONDIMENT**	
		**Quantity**		**Quantity**	
**Breakfast**					
**Snack**					
**Lunch**					
**Sna**					

**Dinner**					
**Snack**					
**Supplements**		*Brand:*		*Dosage:*	

University of Naples "Federico II" - Naples, Italy

Annex 4.

Questionnaires regarding physical activity and sedentary behaviors.

**On how many days in the past week were you physically active for 60 minutes or more?**	**0 days** ☐	**1 days** ☐	**2 days** ☐	**3 days** ☐	**4 days** ☐	**5 days** ☐	**6 days** ☐	**7 days** ☐	☐< **or** ≥☐ **5 times/week**
**Outside school hours, how many hours a week do you usually exercise in your free time so much that you get out of breath or sweat?**	**30 minutes** ☐	**1 hour** ☐	**2-3 hours** ☐	**4-6 hours** ☐	**≥7 hours** ☐	☐< **or** ☐≥ **2 hour/week**			
**About how many hours a day do you usually watch television (including DVDs and videos) in your free time?**	**None at all** ☐	**About 30 min** ☐	**1 hour** ☐	**2 hours** ☐	**Up to ≥7 hour/day** ☐	☐≤ **or >2** ☐ **hour/day**			
**About how many hours a**	**None at all**	**About 30 min**	**1 hour**	**2 hours**	**Up to ≥7 hour/day**	☐≤			

**day do you usually play games on a computer or games console (Playstation, Xbox GameCube etc.) in your free time?**	☐	☐	☐	☐	☐	**or >2** ☐ **hour/day**
**About how many hours a day do you usually use a computer for chatting on-line, internet, emailing, homework etc. in your free time?**	**None at all** ☐	**About 30 min** ☐	**1 hour** ☐	**2 hours** ☐	**Up to ≥7 hour/day** ☐	☐≤ **or >2** ☐ **hour/day**

Ref.: *Haug E, Rasmussen M, Samdal O, Iannotti R, Kelly C, Borraccino A, Vereecken C, Melkevik O, Lazzeri G, Giacchi M, Ercan O, Due P, Ravens-Sieberer U, Currie C, Morgan A, Ahluwalia N; HBSC Obesity Writing Group. Overweight in school-aged children and its relationship with demographic and lifestyle factors: results from the WHO-Collaborative Health Behaviour in School-aged Children (HBSC) study. Int J Public Health. 2009 Sep;54 Suppl 2(Suppl 2):167-79. doi: 10.1007/s00038-009-5408-6. PMID: 19618111; PMCID: PMC2735089.*
