# Supplementary material for: Therapeutic Effects of Butyrate on Pediatric Obesity: A Randomized Clinical Trial
Source: JAMA Netw Open. 2022 Dec 5;5(12):e2244912. doi: 10.1001/jamanetworkopen.2022.44912 (PMC9855301; doi:10.1001/jamanetworkopen.2022.44912)
Supplement: Supplement 2. — eFigure 1. The Distribution of BMI (SDS WHO) at Baseline eFigure 2. The Distribution of HOMA-IR (Unitless) at Baseline eFigure 3. GM Signatures at Baseline Predictable of the Metabolic Response to the Intervention eTable 1. The Baseline Features of Patients Lost to Follow-up eTable 2. The Physical and Sedentary Behaviors Questionnaires Results [file jamanetwopen-e2244912-s002.pdf]

Supplementary Online Content

Coppola S, Nocerino R, Paparo L, et al. Therapeutic effects of butyrate on pediatric obesity: a randomized clinical trial. *JAMA Netw Open*. 2022;5(12):e2244912. doi:10.1001/jamanetworkopen.2022.44912

eFigure 1. The Distribution of BMI (SDS WHO) at Baseline

eFigure 2. The Distribution of HOMA-IR (Unitless) at Baseline

eFigure 3. GM Signatures at Baseline Predictable of the Metabolic Response to the Intervention

eTable 1. The Baseline Features of Patients Lost to Follow-up

eTable 2. The Physical and Sedentary Behaviors Questionnaires Results

This supplementary material has been provided by the authors to give readers additional information about their work.

© 2022 Coppola S et al. *JAMA Network Open.*

eFigure 1. The distribution of BMI (SDS WHO) at baseline

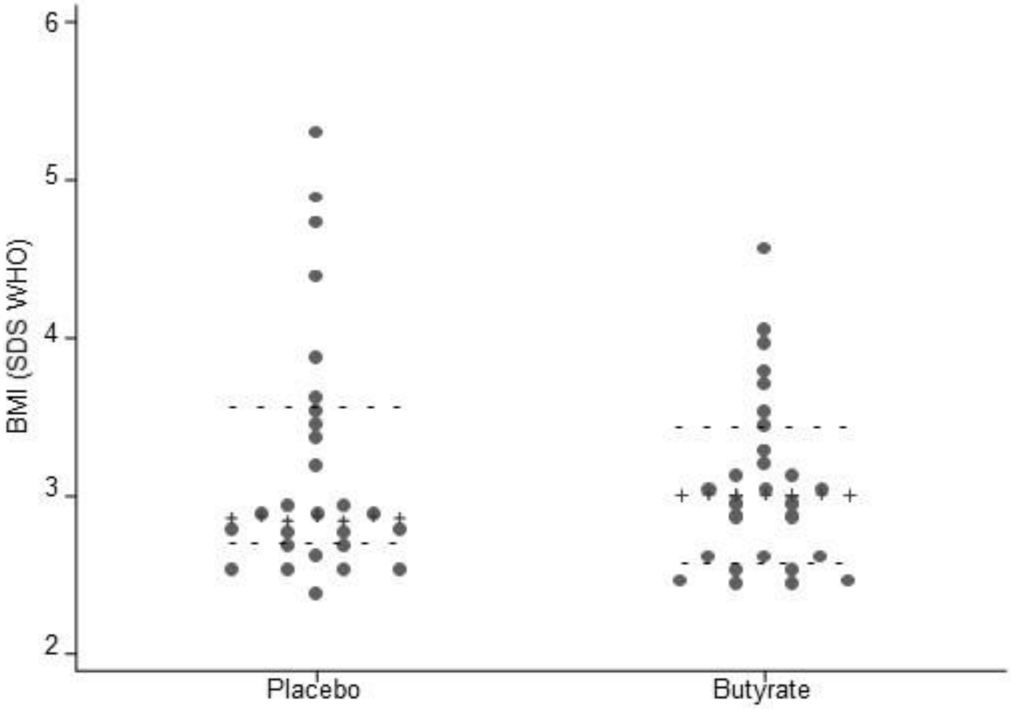

Figure legend: Dotplots showing the distribution of BMI (SDS WHO) at baseline in the placebo and butyrate arms. Horizontal lines are medians and 25th and 75th percentiles.

Abbreviations: BMI: Body Mass Index; SDS: Standard Deviation Score; WHO: World Health Organization

© 2022 Coppola S et al. *JAMA Network Open.*

eFigure 2. The distribution of HOMA-IR (unitless) at baseline

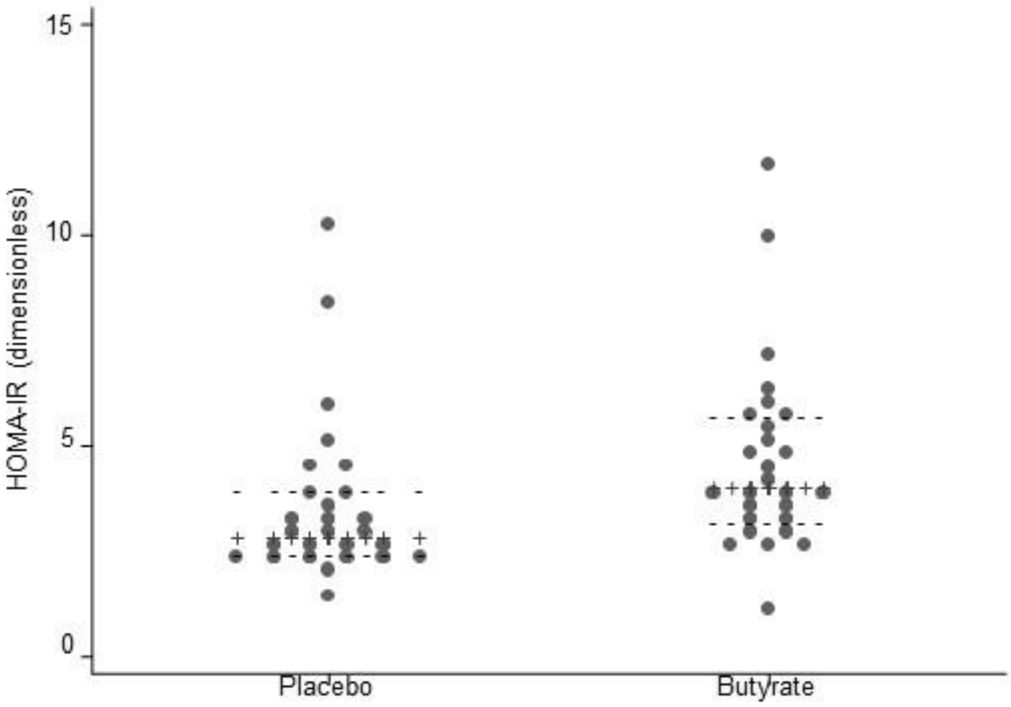

Figure legend: Dotplots showing the distribution of HOMA-IR (unitless) at baseline in the placebo and butyrate arms. Horizontal lines are medians and 25th and 75th percentiles.

Abbreviations: HOMA-IR: Homeostatic Model Assessment of Insulin Resistance.

© 2022 Coppola S et al. *JAMA Network Open.*

eFigure 3. GM signatures at baseline predictable of the metabolic response to the intervention.

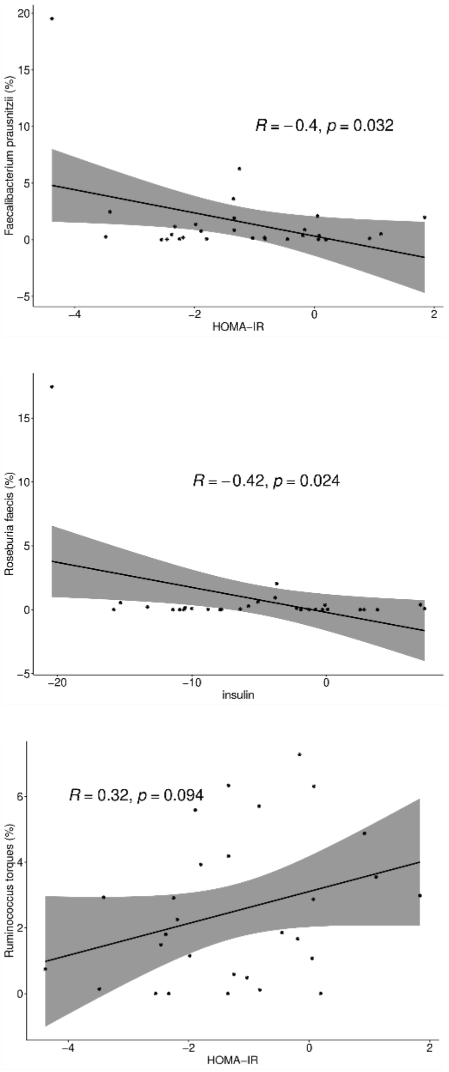

Figure legend: Specific GM signatures at baseline could predict a positive outcome to therapeutic intervention in obese pediatric patients. The baseline abundance of *Faecalibacterium prausnitzii* was positively correlated with the HOMA-IR decrease, the abundance of *Roseburia faecis* was positively correlated with insulin level decrease, while *Ruminococcus torques* showed an opposite trend demonstrating a negative correlation with HOMA-IR levels.

Abbreviations: GM: Gut Microbiome; HOMA-IR: Homeostatic Model Assessment of Insulin Resistance.

© 2022 Coppola S et al. *JAMA Network Open.*

eTable 1. The baseline features of patients lost to follow-up.

	Placebo	Butyrate
	N=2	N=4
Female n (%)	1 (50%)	3 (75%)
Male n (%)	1 (50%)	1 (25%)
Age (years)	9	13 (10 - 14)
Weight (kg)	49.2	70.7 (52.5 - 78.1)
Height (m)	1.41	1.48 (1.39 - 1.57)
BMI (kg/m2)	24.2)	29.6 (26.5 - 32.1)
BMI (SDS WHO)	2.72	2.51 (2.42 - 2.91)
Waist circumference (cm)	70.0	78.2 (76.5 - 82.2)
Glucose (mg/dl)	64	80 (74 - 83)
Insulin (μU/mL)	14	22 (18 - 36)
HOMA-IR (dimensionless)	2.2	4.1 (3.5 - 7.2)
Cholesterol (mg/dl)	174	194 (166 - 206)
LDL-cholesterol (mg/dl)	108	132 (104 - 151)
HDL-cholesterol (mg/dl)	58	50 (45 - 54)
Triglycerides (mg/dl)	67	69 (51 - 88)
Gherlin (pg/ml)	104	175 (147 - 228)
Energy (kcal/day)	2722	3233 (1642 - 4294)
Energy (kcal /kg weight)	53.3	54.9 (31.6 - 64.6)

Figure legend: the table reports the baseline features of patients lost to follow-up. Values are median and IQR (between brackets). IQR cannot be calculated for N = 2.

© 2022 Coppola S et al. *JAMA Network Open.*

eTable 2. The physical activity and sedentary behaviors questionnaires results.

	Placebo Group (n=25)		Butyrate Group (n=23)		
**Items**	**N° of patients**	**% of patients**	**N° of patients**	**% of patients**	***p***
**Moderate to vigorous physical activity ≥ 5 times/week**	15	60	14	60.9	0.951
**Vigorous physical activity ≥ 2 hour/week**	16	64	14	60.9	0.823
**Television-viewing ≤ 2 hour/day**	21	84	20	86.9	0.772
**Electronic game use ≤ 2 hour/day**	20	80	19	82.6	0.817
**Computer use ≤ 2 hour/day**	19	76	18	78.3	0.852

Figure legend: the table reports the results of the questionnaires regarding the physical activity and sedentary behaviors at the end of the study period. Discrete variables are reported as the number and proportion of subjects with the characteristic of interest. The Pearson χ 2 test and the Fischer exact test are applied. The level of significance was 2-sided, $p<0.05$.

© 2022 Coppola S et al. *JAMA Network Open.*
