# Supplementary material for: Therapeutic Effects of Butyrate on Pediatric Obesity: A Randomized Clinical Trial
Source: JAMA Netw Open. 2022 Dec 5;5(12):e2244912. doi: 10.1001/jamanetworkopen.2022.44912 (PMC9855301; doi:10.1001/jamanetworkopen.2022.44912)
Supplement: Supplement 3. — Data Sharing Statement [file jamanetwopen-e2244912-s003.pdf]

Data Sharing Statement

Coppola. Therapeutic Effects of Butyrate Against Pediatric Obesity. *JAMA Netw Open*. Published December 05, 2022. doi:10.1001/jamanetworkopen.2022.44912

Data
Data available: Yes
Data types: Deidentified participant data
How to access data: contact berni@unina.it (corresponding author) to request the data
When available: With publication

Supporting Documents
Document types: Informed consent form
How to access documents: contact berni@unina.it (corresponding author) to request the data
When available: With publication

Additional Information
Who can access the data: anyone requesting the data
Types of analyses: for any purpose
Mechanisms of data availability: with a signed data access agreement
